# Parasite-Induced Th2 Polarization—An Unusual Cause of Paediatric Hepatic Abscess

**DOI:** 10.3390/medicina57121322

**Published:** 2021-12-02

**Authors:** Mara-Ioana Ieşanu, Ramona Cliveti, Mălina Anghel, Mihai-Mirel Stoicescu, Cătălin Boboc, Andreea Ioan, Felicia Galoş

**Affiliations:** 1 Marie Curie Emergency Children’s Hospital, 041451 Bucharest, Romania; ram.doc@yahoo.com (R.C.); malina_malina0@yahoo.com (M.A.); stoima@gmail.com (M.-M.S.); catalin.boboc@hotmail.com (C.B.); berariuandreea@gmail.com (A.I.); felicia_galos@yahoo.com (F.G.); 2 Department of Pediatrics, Carol Davila University of Medicine and Pharmacy, 020021 Bucharest, Romania

**Keywords:** hepatic abscess, parasites, immune system, Th2 response

## Abstract

Liver abscess (LA) is a serious infectious disease, but is relatively rare in the paediatric population, especially in developed countries. Mostly, hepatic abscesses are pyogenic, caused by *Staphylococcus aureus*, while in extremely rare cases can be caused by parasites, such as *Ascaris lumbricoides*. Antimicrobial therapy and percutaneous drainage are the treatments of choice, lowering the mortality caused by this infection. We report a case of a 3-year-old girl admitted to the hospital for abdominal pain and a low-grade fever, with abdominal ultrasonography revealing a hepatic lesion. Initial laboratory tests showed moderate anaemia, thrombocytosis, eosinophilia, high inflammatory markers, and normal liver function. A computed tomography scan revealed two liver abscesses located subdiaphragmatically, and a high immunoglobulin E (IgE) value (22,300 U/mL). After excluding other possible etiologies, the patient was tested for parasitic infections. IgE for *Ascaris lumbricoides* came slightly higher. In addition to empirical antibiotic treatment, the patient received albendazole and made an uneventful recovery, with the full remission of the abscesses and without the need for drainage. In certain cases, parasites such as *Ascaris lumbricoides* are capable of inducing a T helper 2 (Th2) dominated immune response, predisposing the host to eosinophilia, hyperIgE, and increased susceptibility to bacterial infections. Early diagnosis and treatment in these cases may lead to less invasive therapy options in order to obtain a full recovery. To the best of our knowledge, this is the only reported case in the literature of a paediatric patient with parasite-induced liver abscesses, with extremely high IgE values, minimal symptomatology, that made a fast, full recovery without the need of drainage.

## 1. Introduction

Liver abscess (LA) is rarely seen in the paediatric population, especially in developed countries [1]. The incidence rates reported in previous studies vary from 5 to 25 per 100,000 paediatric admissions in developed nations, while in developing ones, they range from 79 to 400 per 100,000 paediatric admissions [2,3,4,5].

In total, 80% of cases consist of pyogenic LA with the most common pathogen being *Staphylococcus aureus* [6]. The evolution of pyogenic LA without treatment can be fatal, but with the appropriate therapy, mortality declines from approximately 40% to less than 15% [6]. *Entamoeba histolytica* is a causative organism, although less than 1% of the infected patients have extraintestinal diseases [7]. Another uncommon cause of LA is the *Ascaris lumbricoides* infection. The prevalence of this infection ranges from 38.4% to 91% in developing countries where sanitation and hygiene are poor [8,9]; however, ascariasis also has a cosmopolitan distribution, featuring cases from developed countries as well [10]. Human ascariasis is usually silent or only manifests as vague stomach symptoms in the majority of affected people. In children, it can cause stunted growth, learning difficulties, and protein-energy and vitamin shortages [11,12]. Although ascariasis is one of the most common parasitic infections in children [13], the LA induced by *Ascaris* is very rare, occurring in less than 1–2% of the cases [14,15]. Moreover, worms present with a distinct mechanism of action and can alter the host immune response, compromising the pathways of immune activation and triggering the stimulation of the T helper 2 (Th2) effector limb with high levels of Immunoglobulin E (IgE) and eosinophilia [16].

Children present a unique set of predisposing factors which make them more vulnerable to parasitic infections, e.g., they have an immature immune system and frequently play in feces-contaminated soil [17]. LA due to parasites is, however, mostly encountered in children who have chronic debilitating conditions, granulocyte malfunction, and congenital or acquired immunosuppression [6].

Herein, we report a case of a parasite-induced hepatic abscess in a 3-year-old previously healthy patient, with important parasite-mediated changes in the immune response.

## 2. Case Presentation

A 3-year-old girl, with no previous pathologic history, who complained of intermittent moderate abdominal pain associated with low-grade fever starting 14 days before, was admitted to the hospital. Regarding her personal background, she had no recent travel history and she came from the rural area.

She was appeared well, with normal vital signs, a benign abdominal exam, but with an abolished vesicular murmur on the right basal pulmonary area.

Initial laboratory tests showed moderate anaemia (Hb = 9.7 g/dL), thrombocytosis (564 × 10^3^/mm^3^), eosinophilia (1.08 × 10^3^/mm^3^), high inflammatory markers (erythrocyte sedimentation rate = 120 mm/h, fibrinogen = 581 mg/dL, C reactive protein = 21.73 mg/dL, ferritin = 257 ng/mL), and normal liver function. Surprisingly, her IgE values were extremely high (22,300 U/mL).

**Figure 1:**
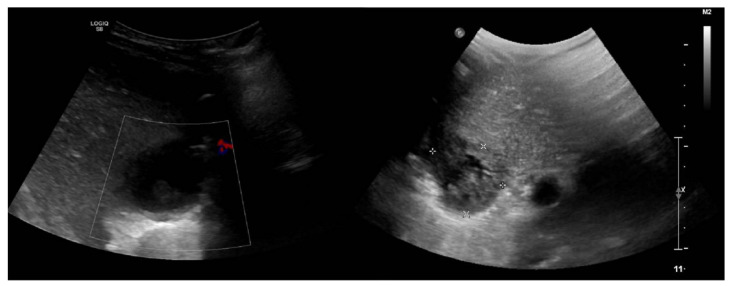
Abdominal ultrasound. Hepatic hypoechoic lesion (approximately 3.5 × 3 cm) with well-defined margins (6 mm) located in the right lobe.

**Figure 2:**
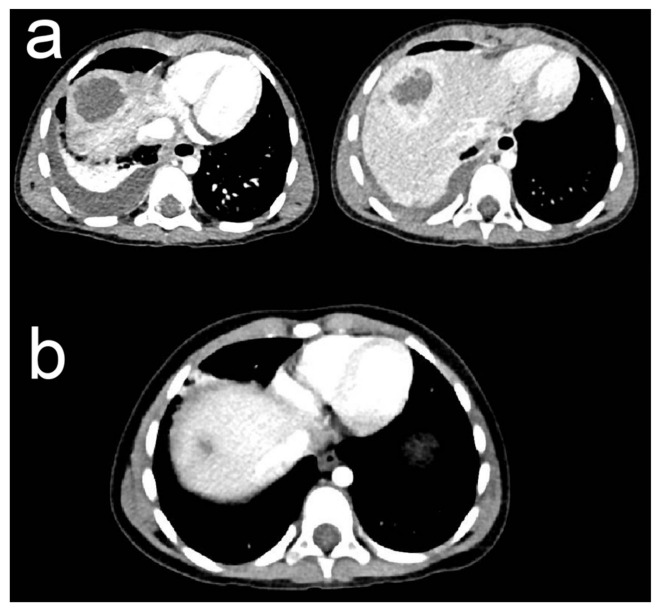
Abdominal Computed Tomography (CT). (**a**) Before treatment—the abdominal CT image showing 2 hypodense collections (size: 39 × 35 × 36 mm and 22 × 20 × 23 mm) on segment VIII of the liver, suggestive of liver abscesses. (**b**) After treatment—the abdominal CT scan reveals the evolution of the hepatic abscesses to a fibrous scar, suggesting the remission of the lesion.

An abdominal ultrasonography (US) initially revealed a 3.5/3 cm heterogeneous hepatic lesion, with a 6 mm wall, with no vascular signal, in a subcapsular location in liver segment VII. Another examination was repeated in our clinic with similar results (Figure 1). To better characterize the hepatic lesion, a thoraco-abdominal computed tomography (CT) was performed. The CT scan revealed two liver collections: peripherally enhanced and centrally hypoattenuating of 39/35/36 mm and 22/20/23 mm, respectively (Figure 2a). These lesions were suggestive of hepatic abscesses. Moreover, they were located subdiaphragmatically in liver segment VIII and spread through the diaphragm into the chest, resulting in moderate right basal pleural effusion and collapse of the right basal pulmonary lobe. The anatomical site of the collections made surgical drainage difficult and it was postponed.

The differential diagnosis included chronic granulomatous disease (CGD), which was excluded after a normal respiratory burst assay. As the alfa-fetoprotein and lactate dehydrogenase levels were normal, malignancy was not considered. Tuberculosis was also excluded as the QuantiFERON test was normal. The blood cultures were negative.

Initially, the patient was placed on intravenous antibiotic therapy with teicoplanin, ceftazidime, and metronidazole. Despite 10 days of antibiotic therapy, the abscess size remained the same. After 2 weeks of hospitalization and antimicrobial drug administration, the patient was discharged, with the recommendation of open surgery drainage in a couple of weeks.

After excluding other possible etiologies, the patient was tested for parasitic infections (*Entamoeba histolytica*, *Toxocara canis*, *Toxoplasma gondii*, *Echinococcus granulosus*, *Ascaris lumbricoides*, *Giardia lamblia*, and *Enterobius vermicularis*). Serological tests were performed for almost all of them, as were Western Blot for *Entamoeba*, *Echinococcus*, and *Toxocara*. The stool examination was negative for *Giardia* fecal antigen, but it revealed cysts of this parasite. The Scotch test did not find any eggs of *Enterobius vermicularis*. IgE for *Ascaris* came back positive, but with low values (0.154 kU/L). Therefore, in addition to empirical antibiotics, following the parasitologist’s recommendation, the patient received a low dose of albendazole (150 mg/day) for 1 month. Inflammatory markers started to decline, as did IgE levels, reaching a value of 1573 U/mL after 2 months; moreover, the eosinophil count returned to normal values (0.55 × 10^3^/mm^3^). A control CT scan after 1 month of albendazole treatment revealed a fibrous linear scar (Figure 2b). The patient made an uneventful recovery, with the full remission of the abscesses.

## 3. Discussion

### 3.1. Hepatic Abscess 

An LA is defined as a puss-filled collection in the liver and is a potentially life-threatening disease. The bulk of these abscesses are classified as pyogenic or amoebic, with parasites and fungi accounting for a small percentage [18].

The incidence of LA has been reported to be 78.9 per 100,000 paediatric admissions in developing countries such as India, while in the USA, the incidence is much lower at approximately 25 per 100,000 paediatric admissions [3,4].

Usually, LAs occur in children with predisposing factors, such as perforated appendicitis, CGD, sickle cell disease, or thalassaemia, and an immunocompromised status due to malignancy or chronic malnutrition [5,19].

The most common etiological agent is *Staphylococcus aureus*, but *Klebsiella pneumoniae* has recently frequently been present, accounting for more than 80% of cases [3,5,6]. Following the pyogenic abscesses, *Entamoeba histolytica* was the next incriminated organism in LA. However, LA due to amebiasis develops in less than 1% of cases [7]. Anaerobes can also contribute to up to 30% of the organisms responsible for LA [19], and fungal infections were reported in children with leukaemia [20]. One study revealed that in almost 36% of cases, the definite cause could not be identified; thus, LA is classified as cryptogenic [21].

In extremely rare cases, parasites, such as *Ascaris lumbricoides*, are incriminated as the causative organisms [22,23,24]. The eggs and larvae are frequently consumed by contaminated water or food, and the *Ascaris lumbricoides*’ constant and strong motions can cause migration into the hepatobiliary system. Ascariasis of the liver can be caused by adult worms and eggs found in the bile ducts or liver parenchyma, or it can also be due to the larvae remaining in the hepatic parenchyma through their life cycle. Biliary worm infection is thought to account for 10–17% of all cases of ascariasis. Biliary colic (56%) is the most common symptom, followed by acute cholangitis (24%), acute cholecystitis (13%), acute pancreatitis (6%), and liver abscesses (less than 1%) [15]. The liver abscess caused by *Ascaris lumbricoides*, in reality, has no distinguishing clinical signs that allow it to be differentiated from abscesses caused by other pathogens.

The clinical signs and symptoms are usually nonspecific and can delay the diagnosis. Patients mostly present with fever, abdominal pain, and nausea and vomiting [5].

Abdominal US is the initial preferred investigation to assess the characteristics of the LA, such as the site, size, and number. Contrast-enhanced CT is more accurate in describing and detecting abscesses anywhere in the liver [19].

After the prompt diagnosis, proper treatment must be initiated. In case of bacterial-induced abscesses, the therapy consists of broad-spectrum antibiotics, which must cover gram-negative, gram-positive, and anaerobic bacteria. If there is a lack of response to medical therapy, with persistent symptoms, sepsis, or enlarging collections, percutaneous drainage (PCD) should be considered. However, if the LA is too close to the pleura, PCD is not indicated, and open surgery must be considered [6]. Nevertheless, when a parasitic infection is suspected, an antiparasitic drug must be added, while the abscess drainage may often be the only therapeutic solution [25].

The modern approach to treating LA with fast diagnostic tools, appropriate antimicrobial therapy, and PCD has remarkably improved the overall survival in paediatric patients.

### 3.2. Case Particularities

The presented case represents a rare complication of parasitic infections, liver abscess, and an extreme Th2 parasite-mediated polarization of the immune system, leading to extremely high values of IgE. The patient had no underlying predisposing factors, was paucisymptomatic considering the clinical findings, and made a complete recovery without invasive treatment.

Firstly, the difficult differential diagnosis included pathologies that could compromise the patient’s immune status. CGD was our main suspicion, especially since this disease can commence with abscesses [26]. CGD is an inherited primary immunodeficiency disease that affects phagocytic function, resulting in impaired protection against bacteria and fungi, leading to recurrent serious life-threatening infections [27].

After we excluded other possible etiologies (i.e., tuberculosis, HIV, malignancy), we looked for parasitic infections. Low levels of *Giardia lamblia* and *Ascaris lumbricoides* were identified. The diagnosis was even more difficult since *Entamoeba histolytica,* the main parasite responsible for LA [28], was negative. Moreover, amebiasis is often encountered in the tropics and subtropics, and given that the patient or relatives had no recent history of traveling, infection with *Entamoeba* was not probable. *Giardia lamblia* infection is self-limiting in immunocompetent individuals. In severe cases, besides affecting the intestinal barrier, it can lead to certain extraintestinal manifestations, such as arthritis and allergic reactions, and even growth and cognitive impairment [29]. Despite these, abscesses were not correlated with this type of infection.

*Ascaris lumbricoides,* one of the most widespread helminthic infections worldwide, has on occasion been implicated in the genesis of abscesses [30]. Therefore, we concluded that our patient’s hepatic lesion might be due to this particular helminth infection.

*Ascaris* is a soil-transmitted helminth that affects humans by infecting them with parasite eggs or larvae that flourish in warm, wet soil. The most common sources of infection in children are contaminated fingers, toys, and soil. The worms leave the human body as eggs and reinfect it as larvae to complete their life cycle. After being triggered by the gastric juice, the eggs hatch in the duodenum, and the resulting rhabditiform larvae travel to the cecum. They pass through the epithelium to arrive to the portal vein, which leads to the liver. Some of them travel to the heart and lungs via the hepatic veins or lymphatics. They reach the bronchial tree after crossing the capillary wall into the alveolar space. During this voyage, they molt twice, ascending to the larynx and hypopharynx before being ingested. They reach sexual maturity in the upper gastrointestinal tract after 2–3 months and molt to become adult worms [31,32]. Dead ova released by female worms may migrate up the common bile duct, causing a granulomatous inflammatory reaction and subsequent disintegration with eosinophil infiltration, resulting in abscesses. The larval stages or ova are more prone to generate inflammation that leads to granulomatous necrosis than adult worms [33].

In general, roundworm infection has a positive prognosis when treated medically, and reinfection is addressed with preventative measures. Management with a combination of medical and interventional treatment, with or without surgery, is the best option for certain biliary tract issues, and presents with a very low or zero death rate and faster reintegration into regular daily life [25].

Adult worms, larvae, or eggs are usually discovered in the abscess or necrotic tissue during surgery, together with a severe inflammatory response and Charcot–Leyden crystals. These crystals consist of eosinophil and basophil derivatives. Their presence implies an inflammatory state, usually caused by allergies or hypersensitivity, with eosinophil and basophil degranulation, which is typical of parasitic infections [34]. Medical treatment must be continued after surgical abscess drainage until the drain is removed, with periodical examinations such as CT scans or US confirming the absence of the abscess.

The suspicion in our patient was a possible infection with the *Ascaris* larvae since we could not even identify the worm by US or CT examination in the abscess. Moreover, the complete resolution of the abscess occurred rapidly following anthelmintic treatment, reinforcing a possible larval state, as an adult worm being difficult to absorb. As a result of the parasitic infection, our patient had moderate eosinophilia, but extremely elevated levels of total IgE, the highest value encountered in our clinic. When these results came back, a hyper-IgE syndrome was initially considered. This is a rare primary immunodeficiency disease, characterized by a classic triad consisting of recurrent bacterial infections of the skin and lung, rash, and IgE values higher than 2000 U/mL [35]. Despite this, our patient had few symptoms, with no pathologic history, no allergic flares, not even pruritus. Moreover, after albendazole treatment, the IgE level decreased significantly.

The initial therapeutic approach in hepatic abscesses is almost always empiric antibiotic treatment and percutaneous drainage, as LAs are difficult to cure. In our patient, the subdiaphragmatic placement of the lesions made the percutaneous technique impossible, so an open surgery was indicated; however, this was postponed for a couple of weeks due to hospital administrative measures. When uncommon etiologic organisms such as parasites are suspected, an antiparasitic drug must be added, although drainage is usually required. The most significant feature of this case was the patient’s excellent response to a long-term low-dose albendazole treatment, which was implemented after consulting a parasitologist and confirming our diagnosis of parasitic infection.

This particular evolution of the patient with the complete uneventful recovery under albendazole treatment suggesting the parasitic etiology, with no invasive therapy needed, along with the extremely high values of IgE representing an exaggerated Th2 immune response, are the main particularities of the case.

### 3.3. Parasitic Infection

Parasites are capable of diminishing the host’s immune system response, and during their transit can switch the immune system from Th1 to Th2 responses, contributing to eosinophilia, hyperIgE, and increased susceptibility to bacterial infections [36,37,38].

T helper cells are lymphocytes that present molecules on their surface known as cluster of differentiation (CD) 4 and are the most prolific cytokine producers. These types of cells divide into two subsets according to the cytokines that they produce: Th1 or Th2, respectively [39]. The entry of pathogens triggers and activates naive Th cells, which differentiate into functional effector cells.

The main Th1 effector cytokines are γ-interferon (γ-IFN) and tumor necrosis factor-α (TNF-α). γ-IFN is responsible for triggering B cells to produce IgG antibodies, which are involved in the phagocytosis of microbes. Both TNF-α and γ-IFN can recruit and activate inflammatory leukocytes, resulting in inflammation and tissue injury [40]. Moreover, γ-IFN can facilitate the differentiation of CD8+ T lymphocytes into active cytotoxic cells [36].

**Figure 3:**
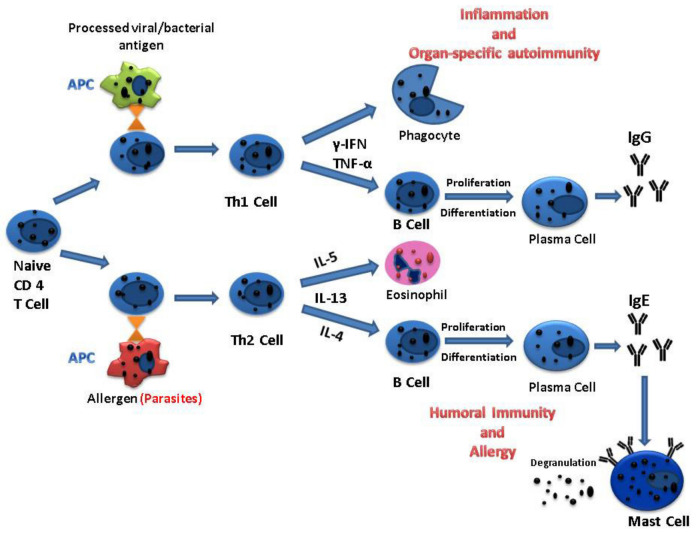
Mechanisms responsible for the two subsets of T helper responses.

Alternatively, the Th2 signature cytokines are interleukin-4 (IL-4), IL-5, and IL-13, which are crucial protection against parasites and in allergic reactions [38,41,42]. IL-4 is capable of instructing B cells to proliferate and differentiate into antibody-secreting plasma cells responsible for IgE production. IgE is capable of triggering the release of mediators by binding to mast cells, which are associated with pruritus [43]. Simultaneously, IL-5, the major eosinophil-activating cytokine, is in charge of the eosinophilic response in parasitic infections (Figure 3).

The delicate balance between Th1 and Th2 responses to an infectious agent plays a central role in host protection. In contrast to intracellular microbes, which determine a Th1 response, extracellular pathogens, such as parasites, typically trigger Th2 responses.

Helminth parasites are the most effective stimulators of IgE biosynthesis currently known. IgE levels in serum can increase 100-fold after a helminth infection, which is proportionately larger than any other immunoglobulin isotype’s reaction [44]. Despite the fact that IgE levels rise during infection, only a small percentage of the serum IgE pool is parasite specific [45]. IgE is involved in the protection of the host against the parasitic agent in helminthic infections, supporting the paradigm that parasitic infections compete for the IgE-Th2 lymphocyte–eosinophil response [38].

In the presented case, the parasitic infection triggered the stimulation of the Th2 effector limb, producing huge amounts of nonspecific IgE along with moderate eosinophilia. The presence of increased IgE production could correspond to chronic allergic symptoms. For example, patients with ascariasis and high IgE levels may experience allergy-like symptoms such as asthma, urticaria, or atopic dermatitis [46], which was not the case in our patient, making the diagnosis more difficult.

In previously reported cases of parasitic infections, IgE levels barely reached approximately 8000 U/mL in a 12-year-old patient suffering from a variety of food allergies, atopic dermatitis, and asthma since infancy [46]. In a cross-sectional study of 101 teenagers from a helminthiasis-endemic area who presented with allergic asthma or rhinitis, the highest identified IgE value in parasitic infections was 1500 U/mL [47]. Our patient, however, presented with remarkable IgE values (22 300 U/mL), which are only found in the reported literature in hyperIgE syndromes [35,48,49]. The confirmation, in our case, of the parasite-induced Th2 polarization was made on the basis of the following: (i) the surprising remission of the hepatic abscess following albendazole treatment, reinforcing the suspicion of parasitic infection; and (ii) the remarkably high levels of IgE and the moderate eosinophilia, which are pivotal traits of the Th2 immune response, values that significantly decreased after the antiparasitic treatment. Although we do not have the imaging confirmation of *Ascaris*, considering the only positive investigation for parasite identification was IgE *Ascaris* specific, and since another diagnosis in this patient could not be established, we concluded that the patient had an *Ascaris*-induced hepatic abscess, with subsequent stimulation of the Th2 effector limb.

In clinical practice, the Th2 immune response demands a high index of suspicion since the exact confirmation test does not exist. In the current case, all the patient’s characteristics and the particular evolution established the Th2 polarization of the immune response induced by the parasitic infection.

## 4. Conclusions

The prevalence of LA in children as a result of parasitic infection is relatively low. The migration of *Ascaris lumbricoides* into the hepatobiliary system, resulting in a hepatic abscess, is a rare complication with just a few cases recorded in the literature, despite the high mortality, the high cost of therapeutic procedures, and lengthy hospitalization involved. This demands a high index of suspicion and the clinical skills to properly diagnose the disease and administer the appropriate therapeutic measures.

The most significant feature of this case was the patient’s excellent response to a long-term low-dose antiparasitic treatment, which confirmed our suspicion of parasitic infection. Furthermore, because no other parasite could be identified, we believe the patient was infected with *Ascaris lumbricoides*. Moreover, we came to the conclusion that our patient had a parasite-induced Th2 polarization based on the exceptionally high IgE levels and eosinophilia, which are the hallmarks of the Th2 immune response.

This case involved a complicated approach, with many differential diagnoses, especially regarding immunodeficiencies. In the end, we believe the patient had a parasite-induced liver abscess with an extreme *Ascaris*-mediated Th2 immune response, which is a common paediatric infection, and she was able to recover entirely without the need for invasive therapy.

## Data Availability

Not applicable.
